# Epidemiology of Peripheral Lymph Node Tuberculosis and Genotyping of *M*. *tuberculosis* Strains: A Case-Control Study

**DOI:** 10.1371/journal.pone.0132400

**Published:** 2015-07-15

**Authors:** Chinmay Khandkar, Zinta Harrington, Peter J. Jelfs, Vitali Sintchenko, Claudia C. Dobler

**Affiliations:** 1 South Western Sydney Clinical School, University of New South Wales, Sydney, New South Wales, Australia; 2 NSW Mycobacterium Reference Laboratory, Centre for Infectious Diseases and Microbiology Laboratory Services, Institute of Clinical Pathology and Medical Research, Westmead Hospital, Sydney, New South Wales, Australia; 3 Marie Bashir Institute for Infectious Diseases and Biosecurity, The University of Sydney, New South Wales, Australia; Singapore Immunology Network, Agency for Science, Technology and Research (A*STAR), SINGAPORE

## Abstract

**Background:**

This study examined potential risk factors of lymph node tuberculosis (LNTB), including phylogenetic lineages of *Mycobacterium tuberculosis* (MTB), in comparison to pulmonary tuberculosis (PTB) in a setting with an ethnically diverse population.

**Methods:**

We conducted a case-control study at a major tuberculosis clinic in Sydney, Australia, which included all patients with peripheral LNTB seen at the clinic between 2000 and 2012. Controls were randomly selected patients with PTB seen at the same clinic during the study period. Epidemiological data were extracted from the hospital electronic database and medical records. Associations between LNTB and age, sex, ethnicity, comorbidities and phylogenetic lineages of MTB in comparison to PTB were examined using logistic regression in univariate and multivariate analyses.

**Results:**

There were 212 cases with LNTB and 424 randomly selected controls with PTB. Among patients with LNTB, 74% were female and the mean age (standard deviation, SD) was 42 (16) years. Among patients with PTB, 43% were female and the mean age was 44 (22) years. Females, 45 to 64-year-olds and Southern Asians had an increased risk for LNTB (OR 3.13, 95% CI 2.10-4.67; OR 2.50, 95% CI 1.29-4.84; OR 3.95, 95% CI 1.54-10.12 respectively). Patients with diabetes were at a higher risk of PTB (OR 0.40, 95% CI 0.19 – 0.83 for LNTB). A subset analysis showed that patients infected with the East African Indian strain of MTB were more likely to develop LNTB (OR 10.07, 95% CI 2.37-42.77).

**Conclusions:**

An increased risk for LNTB (but still lower rates than for PTB) was found among females, people aged 45 to 64 years and people born in Southern Asia. An increased risk for PTB was found among patients with diabetes. The East African Indian strain of MTB was significantly associated with a higher likelihood of LNTB compared to other MTB strains.

## Introduction

The global epidemic of tuberculosis (TB) remains a major health problem worldwide. It has been ranked as the second leading cause of death from an infectious disease after the human immunodeficiency virus (HIV) [1]. In 2011, there were 8.7 million new cases of TB worldwide, and 1.4 million TB deaths [2]. The main form of this disease is pulmonary TB (PTB), which enables transmission of the infection to susceptible hosts. Extra-pulmonary TB (EPTB)—infection of sites other than the lungs—represented 39% of all new TB notifications in Australia in 2011 [3]. Lymph nodes are the second most common site of infection after the lung: they were recorded as a site of infection in 25% of all TB cases and 51% of TB cases with extrapulmonary involvement in the Australian state of New South Wales between 2009 and 2011 [4]. The reasons for post- primary reactivation in the peripheral lymph nodes rather than in the lung are not well understood.

Several factors associated with LNTB have been identified in the literature. Female sex has been shown to have a strong association with LNTB [5–8]. A Californian study showed a female to male ratio of 1.9:1 for incidence of LNTB [6].

Age has also been shown to differ between patient populations with LNTB and PTB. Farer et al. documented a skewed unimodal distribution towards younger age (25–34 years) in LNTB populations, whilst displaying bimodal distribution in their PTB population with peaks at 25–34 years and 65+ years [5]. Studies have also suggested that ethnicity may play a large role. The general consensus drawn from these studies is that LNTB is more prevalent among Asian TB patients compared to other ethnicities [5, 7–9]. Another potential association with LNTB is immunosuppression, as it is well documented that the risk of EPTB increases with immunosuppression [10–12]. Other than host factors, several studies have also analysed the association between certain MTB lineages and the prevalence of EPTB, but have shown conflicting results [13].

This study examined risk factors for LNTB by comparing epidemiological features and genotyping of the causative organisms of peripheral LNTB with PTB in an ethnically diverse population.

## Methods

### Setting and cohort

We conducted a case-control study among a population of LNTB and PTB patients at Liverpool Hospital Chest Clinic, a major TB clinic in Sydney, Australia. Cases were all patients notified with peripheral LNTB between January 2000 and December 2012. Controls were randomly selected (by a computer number generator) from a cohort of patients with PTB notified during the same time period. With two control patients with PTB for each patient with LNTB the study had 80% power to detect an odds ratio (OR) of ≥ 1.8 for the association between a putative risk factor and LNTB. Further increase of the number of controls would not have substantially increased the study power. Patients with more than one disease site (PTB and LNTB combined) were excluded from the study. Patients were identified from the TB notification record book, which contains details of all TB patients who were notified at the TB clinic. For identified participants, patient files were obtained for data extraction.

Epidemiological data were extracted from these files and supplemented by data from the electronic hospital database and the chest clinic database. The data extracted included: gender, age, country of birth and information on co-morbidities (HIV status, diabetes mellitus and chronic kidney disease). Epidemiological data were mapped to records from the New South Wales Mycobacterium reference laboratory, which receives and tests isolates from all bacteriologically confirmed cases in the state. MTB isolates were genotyped by 24-loci Mycobacterial interspersed repetitive unit variable number tandem repeat (MIRU-VNTR) method as previously described and phylogenetic lineages were assigned using the on-line MIRU-VNTR *plus* database [14–16]. The isolates were assigned to global lineages: Lineage 1 –Indo-Oceanic (or East-African-Indian sublineage); Lineage 2 –East Asian (Beijing sublineage); Lineage 3 –East-African-Indian (includes Delhi/Central Asian sublineage) and Lineage 4 –Euro-American (includes Latin-American and Haarlem sublineages). All raw data are available in S1 Dataset.

### Definitions

Countries of birth were categorised according to geographical sub-regions based on the UN M.49 classification scheme [17]. The classification was modified to more appropriately reflect ethnic distribution: Iran was classified with Western Asia (Middle East) rather than Southern Asia. All African sub-regions were grouped into one category ‘Africa’. In similar fashion, all European sub-regions were also combined into a single ‘Europe’ category and Polynesia, Melanesia, and Micronesia were grouped as ‘Pacific Islands’ (see S1 Appendix for countries in each region).

HIV status was determined by examining laboratory reports and clinical files. HIV status was classified as missing if laboratory reports were not present and there was no mention of HIV in clinical notes. HIV testing was not routinely conducted at Liverpool Chest Clinic during the study period, as there is minimal co-infection with HIV and TB in Australia [18]. Patients were assumed to have diabetes and/or chronic kidney disease if the diagnosis was mentioned in the clinical notes or indicated by laboratory results. If there was no mention of diabetes in the clinical notes, or laboratory reports on random/fasting blood glucose levels were normal, we assumed the patient was not diabetic. Patients were assumed to be free of chronic kidney disease if laboratory reports indicated that they had normal creatinine and estimated glomerular filtration rates, with no mention of the disease in clinical notes. All other patients were classified as having missing information.

### Statistical Analysis

Statistical analysis was conducted using SPSS v21.0 (IBM Corp., Armonk, NY, USA). An initial univariate analysis using logistic regression was conducted to estimate ORs with 95% confidence intervals for each identified factor with possible association with LNTB. To control for potential confounders, a multivariate analysis using logistic regression was also conducted to estimate adjusted (independent) ORs with 95% confidence intervals. For the multivariate analysis, only those variables with associated with a p<0.20 in the univariate analysis were included. The geographic regions that provided statistically insignificant results in univariate analysis were grouped together as a single ‘other’ category, to increase study power. Due to missing data for MTB genotyping, a subset multivariate analysis of cases with complete datasets was conducted. Region of birth was excluded as a variable from this analysis to avoid overcorrection, as it is well documented that global lineages of MTB strains vary according to geographical regions.

### Ethical considerations

The study protocol was approved by the South Western Sydney Local Health District Ethics Committee (Reference: HREC/12/LPOOL/445). The requirement for written or verbal patients’ consent for this study was waived by the above ethics committee because existing (clinical) data sources were used. Patient information was anonymized and de-identified prior to analysis.

## Results

The study population included 636 patients in total, of which 212 were cases with LNTB and 424 were randomly selected controls with PTB notified during the same study period. The demographics of both these cohorts are summarised in Table 1. In the LNTB cohort 74% of patients were female compared to 43% among the PTB cohort. The mean age in the LNTB and PTB cohort was similar (42 and 44 years, respectively) as was the median age (40 and 42 years respectively). Although both groups had the peak disease incidence among 25–44 year olds, the LNTB cohort had a higher proportion of cases in this group compared to the PTB cohort (49% vs 35% respectively). Across both cohorts, a majority of patients were from high incidence countries. Patients from Southeast Asia were the biggest group in both cohorts. Patients from Southern Asia were the second biggest group in the LNTB cohort (14% of cohort); in the PTB cohort Australian born patients were the next biggest group (12% of cohort). The PTB cohort had a higher proportion of diabetic patients. Both cohorts had similar distribution patterns for HIV and chronic kidney disease. Genotyping data was available for 295 patients, of which 88 (30%) cases had LNTB, and 207 (70%) cases had PTB. Of all cases infected with East African Indian lineage MTB isolates, 77% (n = 10) were found among patients with LNTB, despite LNTB cases only making up 30% of all patients with genotyping data.

**Table 1:** Patient characteristics and association with LNTB in univariate analysis.

Variable		LNTB cohort (n = 212)	PTB cohort (n = 424)	Crude OR univariate analysis (95% CI)	p Value
Sex, n (%)		212	424		<0.001*
	Female	156 (74%)	182 (43%)	3.72 (2.60–5.31)	
	Male	56 (26%)	242 (57%)	1.00 (reference)	
Age, n (%)		212	424		<0.001*
	0–24	25 (12%)	83 (20%)	1.43 (0.73–2.78)	0.297
	25–44	104 (49%)	150 (35%)	3.33 (1.92–5.79)	<0.001*
	45–64	64 (30%)	100 (24%)	3.07(1.71–5.51)	<0.001*
	65+	19 (9%)	91 (21%)	1.00 (reference)	
Median Age		40	43		
Mean Age		42	44		
Region of Birth, n (%)		209	420		0.002*
	Africa	7 (3%)	15 (3%)	2.08 (0.69–6.31)	0.196
	Southern America	3 (1%)	10 (2%)	1.34 (0.32–5.68)	0.694
	Eastern Asia	6 (3%)	30 (7%)	0.89 (0.30–2.66)	0.836
	Southern Asia	29 (14%)	24 (6%)	5.88 (2.50–13.84)	<0.001*
	South East Asia	135 (65%)	253 (60%)	2.41 (1.21–4.79)	0.012*
	Western Asia	5 (2%)	8 (2%)	2.22 (0.63–7.83)	0.212
	Europe	3 (1%)	15 (4%)	0.89 (0.22–3.62)	0.872
	Pacific Islands	10 (5%)	17 (4%)	2.23 (0.76–6.50)	0.143
	Australia & New Zealand	11 (5%)	48 (12%)	1.00 (reference)	
HIV status, n (%)		55	110		
	HIV positive	3 (5%)	3 (3%)	2.06 (0.40–10.55)	0.387
	HIV negative	52 (95%)	107 (97%)	1.00 (reference)	
Diabetes, n (%)		176	401		0.006*
	Has Diabetes	11 (6%)	58 (14%)	0.38 (0.19–0.76)	
	No Diabetes	165 (94%)	343 (86%)	1.00 (reference)	
Chronic Kidney Disease, n (%)		129	345		0.44
	Has CKD	3 (2%)	13 (4%)	0.61 (0.17–2.16)	
	No CKD	126 (98%)	332 (96%)	1.00 (reference)	
MTB lineages, n (%)		88	207		0.014*
	Indo-Oceanic	32 (36%)	78 (38%)	1.09 (0.62–1.94)	0.759
	Euro American	13 (15%)	38 (18%)	0.91 (0.43–1.92)	0.809
	East African Indian	10 (11%)	3 (1%)	9.89 (2.30–34.42)	0.002*
	East Asian	33 (38%)	88 (43%)	1.00 (reference)	

In univariate analysis (Table 1), females were almost four times more likely to have LNTB compared to males (OR 3.72, 95% CI 2.60–5.31, p < 0.001). Patients aged 24 to 44 years had an OR of 3.33 (95% CI 1.92–5.79, p < 0.001) for LNTB compared to patients 65 years or older (p < 0.001). Middle-aged patients (44 to 65 years old) were also significantly more likely to present with LNTB compared to older patients (OR 3.07, 95% CI 1.71–5.51, p < 0.001).

Multivariate analysis included variables associated with a p < 0.20 in the univariate analysis (Table 2). Thus, the potential confounders corrected for in the multivariate analysis included gender, age, geographic origin and diabetes status. A subset multivariate analysis was conducted for patients with complete datasets for MTB genotyping (Table 3).

**Table 2:** Patient characteristics and associations with LNTB in multivariate analysis.

Variable		LNTB cohort (n = 176)	PTB cohort (n = 401)	Adjusted OR multivariate analysis (95% CI)	p Value
Sex, n (%)					<0.001*
	Female	125 (71%)	173 (43%)	3.13 (2.10–4.67)	
	Male	51 (29%)	228 (57%)	1.00 (reference)	
Age, n (%)					0.006*
	0–24	22 (12%)	79 (20%)	0.93 (0.43–2.02)	0.855
	25–44	84 (48%)	144 (36%)	1.78 (0.93–3.39)	0.082
	45–64	54 (31%)	93 (23%)	2.50 (1.29–4.84)	0.007*
	65+	16 (9%)	85 (21%)	1.00 (reference)	
Diabetes, n (%)					0.014*
	History of Diabetes	11 (6%)	58 (14%)	0.40 (0.19–0.83)	
	No Diabetes	165 (94%)	343 (86%)	1.00 (reference)	
Region of Birth, n (%)					0.009*
	Southern Asia	28 (16%)	23 (6%)	3.95 (1.54–10.12)	0.004*
	South East Asia	106 (60%)	236 (59%)	1.49 (0.70–3.20)	0.301
	Others	31 (18%)	94 (23%)	1.27 (0.55–2.93)	0.575
	Australia & New Zealand	11 (6%)	48 (12%)	1.00 (reference)	

**Table 3:** Subset multivariate analysis including cases with MTB genotyping, associations with LNTB in multivariate analysis.

Variable		LNTB cohort (n = 80)	PTB cohort (n = 200)	Adjusted OR multivariate analysis (95% CI)	p Value
Sex, n (%)					<0.001*
	Female	59 (74%)	83 (42%)	3.73 (2.03–6.86)	
	Male	21 (26%)	117 (58%)	1.00 (reference)	
Age, n (%)					0.252
	0–24	7 (9%)	25 (13%)	0.64 (0.20–2.05)	0.449
	25–44	38 (48%)	76 (38%)	1.40 (0.60–3.29)	0.442
	45–64	25 (31%)	53 (26%)	1.70 (0.70–4.15)	0.224
	65+	10 (12%)	46 (23%)	1.00 (reference)	
Diabetes, n (%)					0.130
	History of Diabetes	4 (5%)	29 (15%)	0.39 (0.12–1.32)	
	No Diabetes	76 (95%)	171 (85%)	1.00 (reference)	
MTB lineages, n (%)					0.018*
	Indo Oceanic	27 (34%)	75 (38%)	1.19 (0.62–2.27)	0.602
	Euro American	13 (16%)	37 (18%)	1.00 (0.45–2.23)	1.000
	East African Indian	10 (13%)	3 (2%)	10.07 (2.37–42.77)	0.002*
	East Asian	30 (37%)	85 (42%)	1.00 (reference)	

The multivariate analysis included 577 cases, of which 401 were patients with PTB and 176 were patients with LNTB. The demographic characteristics of included cases and controls are summarised in Table 2. All included variables had an overall significant association with LNTB in multivariate analysis (for the main groups). Females were still 3 times more likely to have LNTB than males (95% CI 2.10–4.67, p < 0.001). Unlike in the univariate analysis however, when examining age subgroups, only those in the 45 to 64 year old age bracket were at significantly higher risk for LNTB compared to the older population (OR 2.50, 95% CI 1.29–4.84, p = 0.007). Furthermore only the subgroup born in Southern Asia was at an increased risk of LNTB (OR 3.95 95% CI 1.54–10.12, p = 0.004). There was no significantly increased risk of LNTB for those from South East Asia (p = 0.301). Finally those with diabetes were still significantly less likely to develop LNTB (OR 0.40 95% CI 0.19–0.83, p = 0.014).

The subset analysis included 280 patients with complete data including information on genotyping of MTB. (Table 3). The MTB East African Indian lineage was significantly associated with LNTB (OR 10.07, 95% CI 2.37–42.77, p = 0.002).

## Discussion

This study significantly expanded previous observations that phylogenetic subgroups of MTB can be associated with different clinical manifestations [13, 19, 20]. In this case control study, we identified several factors associated with LNTB. The MTB East African Indian lineage was significantly associated with LNTB (OR 10.07, 95% CI 2.37–42.77, p = 0.002). Being female, middle-aged (44 to 65 years), and born in Southern Asia was significantly associated with LNTB. Patients with diabetes mellitus had increased susceptibility to PTB.

The East-Asian MTB strain dominated both LNTB and PTB cohorts, a reflection of the large Southeast Asian population in our study sample. The East African Indian lineage had an OR of 10.07 to be associated with LNTB compared to other strains. This differed in a study by Firdessa et al. that found identical lineage distribution across both cohorts [21]. While this result could possibly indicate that the East African Indian strain has unique virulence factors, it may also be a reflection of the higher prevalence of LNTB in the South Asian population from our study. This is because the East African Indian strain is the most prevalent genotype causing EPTB in this region [22].

The South East Asian population dominated both cohorts- LNTB and PTB, with Southern Asians making up the second and third largest proportion in the LNTB and PTB cohort respectively. It has been documented in the literature that Asians are more predisposed to LNTB compared to non-Asians. A previous Australian study found that Asian migrants were over-represented among patients with LNTB, making up 71% of all patients with LNTB [23]. A similar result was found in a large American multi centre study [8]. These studies did not differentiate between the subregions in Asia with a diverse range of ethnicities. In our study, only people from Southern Asia were significantly predisposed to LNTB in multivariate analysis.

**Fig 1:**
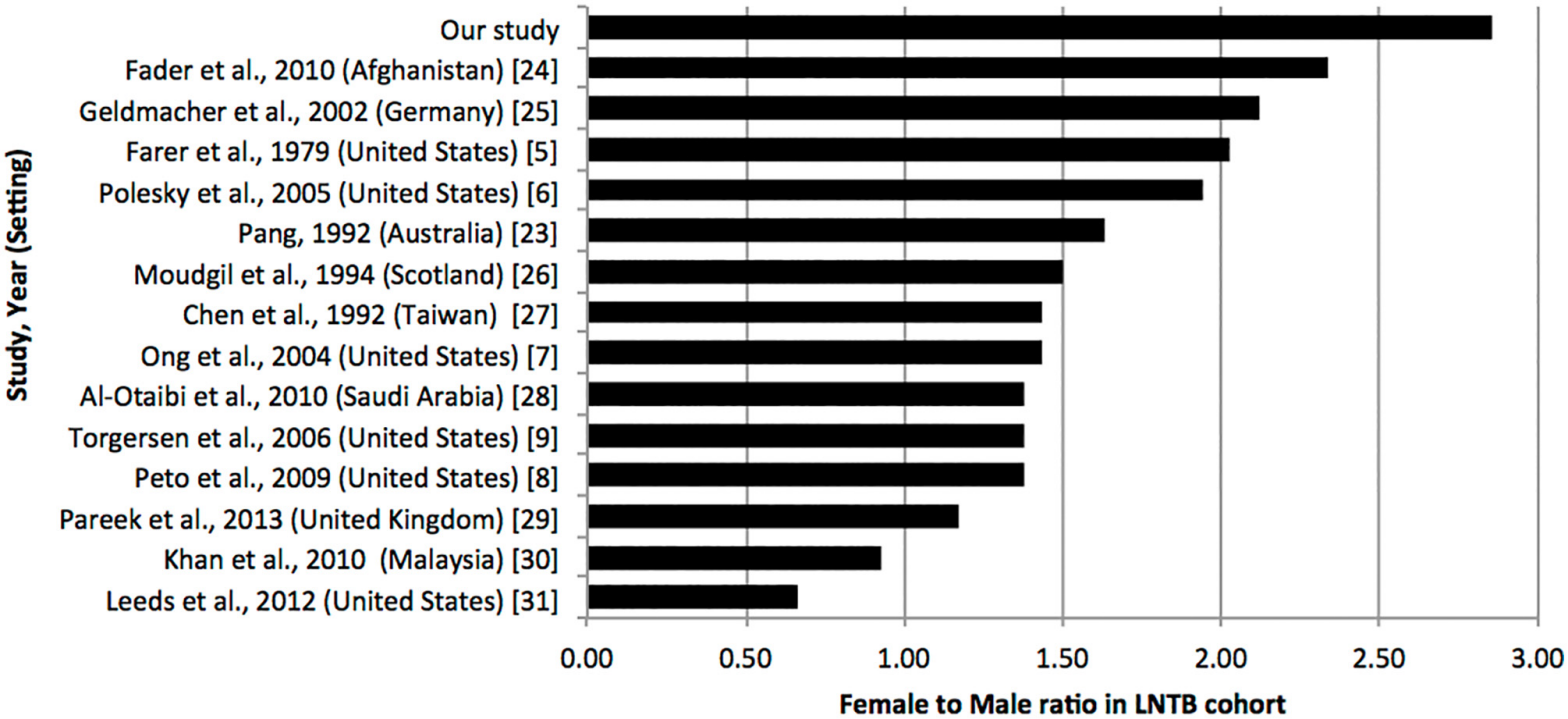
Ratio of females to males in LNTB cohorts of published studies.

The female to male ratio for LNTB was 2.8:1, which is consistent with previous studies that found that LNTB is more common among women (Fig 1) [5–9, 23–31]. Our study showed that females had an adjusted OR of 3.13 for LNTB compared with males, similar to a previous study that showed an adjusted OR of 3.5 [7]. In our study there was a higher female to male ratio (2.8:1) compared with other studies, in which the highest reported ratio was 2.3:1 [24]. The reason for the association of female sex with LNTB is not well understood. One study found a difference in tumour necrosis factor and interleukin-10 production between both sexes, thus suggesting that this difference may play a role in susceptibility to LNTB [32]. Other suggested factors to explain this difference between sexes include CD4+ lymphocyte counts, endocrine factors, socio-economic factors and cultural factors [13, 33]. A possible reason why males dominated the PTB cohort is because males are more likely to be smokers, which has been well documented to increase the risk of PTB [34]. However, a Taiwanese study found that females had an increased risk for EPTB compared to males (OR 1.69, 95% CI 1.02–2.80, p = 0.04) even when correcting for smoking as potential confounding factor (OR 0.57 for EPTB, 95% CI 0.34–0.95, p = 0.03) [35]. Hence it is possible that females are at a higher risk of LNTB compared to males, independent of smoking status.

The peak incidence of LNTB was among people aged 25–44 years, which made up almost half of the LNTB cohort (49%). This is consistent with previous findings, which showed that the peak incidence occurs between 30–40 years of age [8, 30, 31]. The average age for Australian born patients with peripheral LNTB in our study was 29 years, whilst the average age in the remaining LNTB cohort (of which 99% were born in a developing country) was 43 years. This was surprising, as studies in both Germany and Scotland have shown that locally born patients with LNTB are older compared to immigrants [25, 26]. The reasons for this discrepancy are unclear.

It has been well documented that TB is more common among persons with an impaired host defence [36]. A study from Japan showed that serum levels of Th-1 related cytokines, which play a central role in host defence to mycobacterial infection, were reduced in diabetic patients [37]. Our multivariate analysis indicated that diabetes mellitus was significantly more prevalent in the PTB cohort compared to the LNTB cohort. This is consistent with previous reports, which suggest that diabetes could be a significant risk factor for PTB, but not for EPTB [38–40].

## Conclusions

This case-control study found that the East African Indian strain of MTB was significantly associated with LNTB. Further, female sex, age between 45 to 64 years, and Southern Asia origin were associated with an increased risk for LNTB. In contrast, diabetes mellitus was found to have a stronger association with PTB. These observations improve our understanding of risk factors for EPTB.

## Supporting Information

S1 Appendix Modified M.49 UN classification scheme. (PDF)

S1 Dataset Data of all TB patients. (PDF)
