# Supplementary material for: Epidemiology of Peripheral Lymph Node Tuberculosis and Genotyping of M. tuberculosis Strains: A Case-Control Study
Source: PLoS One. 2015 Jul 15;10(7):e0132400. doi: 10.1371/journal.pone.0132400 (PMC4503442; doi:10.1371/journal.pone.0132400)
Supplement: S1 Appendix — (PDF) [file pone.0132400.s001.pdf]

Appendix A: Modified M.49 UN classification scheme

Adapted from the original M.49 UN classification scheme [17].

Africa
Algeria
Angola
Benin
Botswana
Burkina Faso
Burundi
Cameroon
Cape Verde
Central African Republic
Chad
Comoros
Congo
Cote d'Ivoire
Democratic Republic of the Congo
Djibouti
Egypt
Equatorial Guinea
Eritrea
Ethiopia
Gabon
Gambia
Ghana
Guinea
Guinea-Bissau
Kenya
Lesotho
Liberia
Libya
Madagascar
Malawi
Mali
Mauritania
Mauritius
Mayotte
Morocco
Mozambique
Namibia
Niger
Nigeria
Réunion
Rwanda
Saint Helena
Sao Tome and Principe
Senegal
Seychelles
Sierra Leone
Somalia
South Africa
South Sudan
Sudan
Swaziland
Togo
Tunisia
Uganda
United Republic of Tanzania
Western Sahara
Zambia
Zimbabwe
Caribbean
Anguilla
Antigua and Barbuda
Aruba
Bahamas
Barbados

Bonaire, Saint Eustatius and Saba
British Virgin Islands
Cayman Islands
Cuba
Curaçao
Dominica
Dominican Republic
Grenada
Guadeloupe
Haiti
Jamaica
Martinique
Montserrat
Puerto Rico
Saint-Barthélemy
Saint Kitts and Nevis
Saint Lucia
Saint Martin (French part)
Saint Vincent and the Grenadines
Sint Maarten (Dutch part)
Trinidad and Tobago
Turks and Caicos Islands
United States Virgin Islands
Central America
Belize
Costa Rica
El Salvador
Guatemala
Honduras
Mexico
Nicaragua
Panama
South America
Argentina
Bolivia (Plurinational State of)
Brazil
Chile
Colombia
Ecuador
Falkland Islands (Malvinas)
French Guiana
Guyana
Paraguay
Peru
Suriname
Uruguay
Venezuela (Bolivarian Republic of)
Northern America b/
Bermuda
Canada
Greenland
Saint Pierre and Miquelon
United States of America
Central Asia
Kazakhstan
Kyrgyzstan
Tajikistan
Turkmenistan
Uzbekistan
Eastern Asia
China
China, Hong Kong Special Administrative Region
China, Macao Special Administrative Region
Democratic People's Republic of Korea
Japan
Mongolia
Republic of Korea

Southern Asia
Afghanistan
Bangladesh
Bhutan
India
Maldives
Nepal
Pakistan
Sri Lanka
South-Eastern Asia
Brunei Darussalam
Cambodia
Indonesia
Lao People's Democratic Republic
Malaysia
Myanmar
Philippines
Singapore
Thailand
Timor-Leste
Viet Nam
Western Asia
Armenia
Azerbaijan
Bahrain
Cyprus
Georgia
Iran
Iraq
Israel
Jordan
Kuwait
Lebanon
Oman
Qatar
Saudi Arabia
State of Palestine
Syrian Arab Republic
Turkey
United Arab Emirates
Yemen
Europe
Åland Islands
Albania
Andorra
Austria
Belarus
Belgium
Bosnia and Herzegovina
Bulgaria
Channel Islands
Croatia
Czech Republic
Denmark
Estonia
Faeroe Islands
Finland
France
Germany
Gibraltar
Greece
Guernsey
Holy See
Hungary
Iceland
Ireland
Isle of Man
Italy

Jersey
Latvia
Liechtenstein
Lithuania
Luxembourg
Malta
Monaco
Montenegro
Netherlands
Norway
Poland
Portugal
Republic of Moldova
Romania
Russian Federation
San Marino
Sark
Serbia
Slovakia
Slovenia
Spain
Svalbard and Jan Mayen Islands
Sweden
Switzerland
Ukraine
United Kingdom of Great Britain and Northern Ireland
The former Yugoslav Republic of Macedonia
Australia and New Zealand
Australia
New Zealand
Norfolk Island
Pacific Islands
American Samoa
Cook Islands
Fiji
French Polynesia
Guam
Kiribati
Marshall Islands
Micronesia (Federated States of)
Nauru
New Caledonia
Niue
Northern Mariana Islands
Palau
Papua New Guinea
Pitcairn
Samoa
Solomon Islands
Tokelau
Tonga
Tuvalu
Vanuatu
Wallis and Futuna Islands
