# Supplementary material for: Epidemiology of Peripheral Lymph Node Tuberculosis and Genotyping of M. tuberculosis Strains: A Case-Control Study
Source: PLoS One. 2015 Jul 15;10(7):e0132400. doi: 10.1371/journal.pone.0132400 (PMC4503442; doi:10.1371/journal.pone.0132400)
Supplement: S1 Dataset — (PDF) [file pone.0132400.s002.pdf]

Case number	Disease site	Gender	Age group	BCG	Smoking	CKD	HIV	diabetes	UN regions	Modified UN region	Genotype
1	0	0	3	missing	missing	0	missing	0	1	3	3
2	0	1	3	missing	missing	missing	missing	missing	5	2	missing
3	0	0	3	1	0	0	0	0	6	3	4
4	0	0	3	missing	1	0	missing	0	3	3	2
5	0	1	3	missing	missing	0	missing	0	5	2	2
6	0	0	3	missing	0	missing	missing	0	3	3	missing
7	0	1	3	missing	0	0	missing	0	3	3	1
8	0	0	3	missing	1	missing	missing	1	3	3	missing
9	0	0	3	0	missing	1	missing	0	5	2	2
10	0	1	3	missing	missing	0	missing	0	3	3	1
11	1	1	3	missing	missing	missing	missing	missing	5	2	missing
12	0	0	3	missing	missing	missing	missing	missing	3	3	missing
13	0	0	3	missing	1	0	missing	0	5	2	missing
14	0	1	3	1	missing	0	missing	0	7	3	3
15	0	1	3	missing	missing	0	missing	0	5	2	missing
16	0	0	3	1	0	0	missing	0	5	2	1
17	0	0	3	missing	missing	0	0	1	5	2	missing
18	0	0	3	missing	missing	0	missing	0	3	3	2
19	0	0	3	missing	1	1	missing	1	5	2	2
20	0	0	3	missing	missing	0	missing	0	3	3	1
21	1	1	3	0	missing	missing	missing	0	5	2	1
22	0	1	3	missing	missing	0	missing	0	missing	3	missing
23	0	0	3	missing	missing	1	missing	1	2	3	missing
24	0	0	3	missing	0	0	missing	0	3	3	2
25	0	0	3	missing	missing	0	missing	1	5	2	2
26	0	1	3	missing	missing	missing	missing	missing	2	3	missing
27	0	0	3	0	1	0	missing	0	7	3	1
28	1	0	3	missing	missing	0	missing	0	5	2	missing
29	0	0	3	missing	missing	missing	missing	missing	5	2	missing
30	0	0	3	missing	1	1	missing	0	5	2	1
31	1	1	3	missing	missing	missing	missing	missing	4	1	missing

32	1	0	3	missing	1	0	0	0	5	2	1
33	0	0	3	missing	1	0	missing	0	5	2	1
34	0	1	3	missing	1	1	missing	0	5	2	missing
35	0	0	3	missing	missing	1	missing	0	5	2	3
36	0	1	3	missing	0	missing	missing	0	0	0	missing
37	1	0	3	missing	missing	missing	missing	0	5	2	missing
38	0	1	3	missing	0	0	missing	1	5	2	1
39	0	1	3	missing	missing	0	missing	1	5	2	1
40	0	0	3	missing	1	1	missing	1	missing	3	missing
41	0	0	3	missing	0	0	missing	0	5	2	missing
42	0	1	3	missing	missing	0	missing	0	5	2	1
43	0	0	3	1	0	0	missing	0	4	1	missing
44	0	0	3	missing	0	0	missing	1	4	1	1
45	0	1	3	0	0	0	missing	1	5	2	missing
46	0	1	3	missing	missing	missing	missing	missing	7	3	3
47	0	0	3	0	missing	missing	missing	0	3	3	1
48	1	0	3	missing	missing	0	missing	1	5	2	3
49	0	0	3	missing	0	missing	missing	0	7	3	1
50	0	0	3	0	missing	0	missing	0	5	2	2
51	0	0	3	missing	1	0	missing	0	2	3	missing
52	0	0	3	0	0	0	0	0	3	3	2
53	0	0	3	0	1	0	0	1	5	2	2
54	0	0	3	missing	missing	0	missing	0	0	0	missing
55	0	0	3	missing	1	0	missing	1	3	3	missing
56	0	0	3	0	missing	missing	missing	0	0	0	missing
57	0	0	3	missing	0	0	0	0	5	2	missing
58	1	1	3	missing	missing	1	missing	1	5	2	missing
59	0	1	3	missing	missing	0	missing	1	5	2	missing
60	1	0	3	missing	0	0	missing	0	5	2	missing
61	0	0	3	missing	1	0	0	0	5	2	missing
62	0	1	3	missing	missing	0	missing	1	5	2	1
63	0	0	3	missing	0	0	missing	0	5	2	1
64	0	1	3	missing	missing	missing	missing	0	3	3	missing

65	0	1	3	missing	missing	0	missing	1	5	2	2
66	0	0	3	0	missing	missing	missing	0	5	2	2
67	0	0	3	0	1	0	0	1	5	2	1
68	1	1	3	missing	missing	missing	missing	missing	5	2	missing
69	0	0	3	missing	missing	missing	missing	missing	5	2	1
70	0	0	3	missing	0	0	missing	0	5	2	2
71	0	0	3	missing	0	0	missing	0	5	2	1
72	0	0	3	0	1	0	missing	0	5	2	missing
73	0	1	3	missing	missing	1	missing	1	5	2	missing
74	1	0	3	missing	1	0	missing	0	5	2	missing
75	0	1	3	1	missing	0	missing	0	5	2	missing
76	0	0	3	1	1	0	missing	0	5	2	missing
77	0	0	3	missing	1	0	0	0	3	3	1
78	0	0	3	missing	1	0	missing	1	5	2	2
79	0	0	3	missing	missing	0	missing	1	8	3	missing
80	0	1	3	missing	0	0	0	1	4	1	4
81	0	1	3	0	0	0	missing	0	5	2	missing
82	0	0	3	missing	1	0	missing	1	5	2	missing
83	0	1	3	missing	0	0	missing	1	3	3	missing
84	1	1	3	0	0	0	missing	0	5	2	1
85	1	1	2	missing	missing	missing	missing	missing	5	2	missing
86	0	0	3	missing	1	missing	missing	0	4	1	4
87	0	1	3	0	missing	missing	missing	0	0	0	missing
88	1	1	2	missing	missing	missing	missing	missing	1	3	1
89	0	1	3	missing	0	0	missing	1	8	3	3
90	1	1	3	missing	0	0	missing	0	0	0	1
91	0	0	3	0	missing	missing	missing	0	7	3	missing
92	0	0	3	missing	missing	0	missing	0	0	0	2
93	0	0	2	missing	missing	missing	missing	missing	5	2	missing
94	0	0	3	missing	1	0	missing	0	5	2	1
95	1	1	3	1	0	0	missing	0	5	2	2
96	1	0	2	1	missing	missing	missing	0	4	1	missing
97	0	0	3	0	1	0	0	1	5	2	2

98	0	1	3	0	missing	0	missing	0	5	2	2
99	0	0	3	missing	missing	0	missing	1	4	1	1
100	0	0	3	missing	missing	missing	missing	1	3	3	1
101	0	1	2	missing	0	0	missing	0	5	2	missing
102	0	0	3	missing	missing	0	missing	0	0	0	missing
103	1	1	3	missing	0	0	missing	0	7	3	3
104	1	1	3	1	1	missing	missing	0	0	0	3
105	0	1	2	missing	missing	missing	missing	missing	5	2	missing
106	0	0	3	missing	1	0	missing	0	5	2	1
107	1	1	3	missing	missing	0	0	1	5	2	2
108	0	1	3	missing	1	0	missing	1	5	2	missing
109	0	0	2	missing	1	0	missing	0	0	0	missing
110	1	1	2	missing	missing	1	missing	1	5	2	missing
111	0	0	3	0	1	0	0	0	5	2	3
112	0	0	3	0	missing	missing	missing	1	5	2	2
113	1	0	2	missing	missing	missing	missing	0	5	2	missing
114	0	0	3	missing	1	0	0	0	6	3	3
115	0	1	2	1	0	1	0	1	4	1	2
116	0	0	3	missing	1	0	missing	0	5	2	3
117	1	1	3	missing	missing	0	missing	0	3	3	1
118	0	0	2	missing	missing	missing	missing	missing	5	2	missing
119	0	0	2	missing	missing	0	missing	0	3	3	missing
120	1	1	2	missing	missing	missing	missing	0	6	3	missing
121	0	0	2	missing	missing	missing	missing	missing	4	1	2
122	1	0	2	1	1	0	missing	0	5	2	missing
123	0	0	3	missing	missing	0	missing	0	7	3	missing
124	0	0	2	missing	missing	0	missing	0	5	2	missing
125	0	1	2	0	0	0	missing	0	7	3	3
126	0	1	3	missing	missing	0	missing	0	0	0	1
127	1	1	2	missing	missing	missing	missing	missing	7	3	missing
128	0	0	2	missing	missing	missing	missing	1	8	3	missing
129	1	1	3	0	1	0	missing	0	2	3	3
130	0	0	2	missing	missing	0	missing	0	5	2	1

131	0	0	2	missing	1	0	missing	1	5	2	1
132	0	0	2	missing	1	0	missing	1	5	2	2
133	0	0	2	missing	1	0	missing	1	8	3	3
134	0	0	2	missing	1	0	missing	0	5	2	1
135	1	1	2	1	0	0	missing	0	5	2	1
136	1	1	2	missing	missing	missing	missing	missing	5	2	missing
137	0	0	2	1	1	0	missing	0	5	2	2
138	1	1	2	0	0	0	missing	1	5	2	missing
139	0	0	2	missing	missing	missing	missing	0	0	0	missing
140	0	1	2	missing	missing	0	0	0	5	2	missing
141	0	0	2	missing	1	0	missing	1	8	3	1
142	1	1	2	missing	missing	missing	missing	missing	5	2	missing
143	0	1	2	missing	missing	0	missing	0	5	2	3
144	0	1	2	1	0	0	missing	1	4	1	4
145	0	0	2	missing	missing	0	missing	1	5	2	1
146	1	0	2	missing	missing	0	0	0	5	2	2
147	1	1	2	missing	0	1	missing	0	5	2	missing
148	0	1	2	missing	0	0	0	0	5	2	2
149	0	0	2	missing	1	0	missing	1	5	2	missing
150	1	1	2	0	missing	missing	missing	0	5	2	missing
151	0	0	2	0	1	missing	missing	0	5	2	missing
152	0	0	2	missing	1	0	missing	0	7	3	1
153	0	0	2	0	1	0	missing	0	3	3	2
154	0	0	2	missing	missing	0	missing	0	5	2	2
155	0	1	2	missing	0	0	0	1	5	2	1
156	1	1	2	1	missing	missing	missing	0	7	3	missing
157	1	1	2	1	missing	0	0	0	5	2	2
158	1	1	2	1	0	0	missing	0	5	2	missing
159	1	0	2	1	missing	0	missing	0	5	2	missing
160	1	0	2	missing	0	0	0	0	0	0	2
161	1	1	2	1	1	0	missing	0	3	3	3
162	1	1	2	missing	0	0	0	0	5	2	missing
163	0	0	2	missing	1	0	0	1	5	2	2

164	0	0	2	missing	1	0	0	1	5	2	1
165	0	0	2	missing	missing	missing	missing	missing	5	2	missing
166	0	0	2	missing	missing	0	missing	0	6	3	missing
167	0	1	2	1	0	0	missing	0	1	3	missing
168	0	0	2	0	missing	0	missing	0	5	2	3
169	0	0	2	1	1	missing	missing	1	8	3	4
170	1	0	2	1	0	0	0	1	4	1	1
171	0	0	2	missing	missing	0	missing	0	missing	3	missing
172	0	0	2	0	missing	missing	missing	0	5	2	2
173	1	1	2	missing	0	missing	missing	0	5	2	1
174	0	1	2	missing	0	0	missing	0	1	3	missing
175	0	0	2	0	1	0	missing	1	5	2	1
176	1	1	2	missing	0	missing	0	0	8	3	4
177	0	0	2	1	1	0	0	0	5	2	2
178	0	0	2	missing	1	0	0	1	5	2	2
179	0	1	2	1	0	0	0	0	5	2	4
180	1	1	2	1	missing	0	0	0	8	3	missing
181	1	1	2	missing	missing	0	missing	0	5	2	3
182	0	1	2	1	0	0	1	0	1	3	missing
183	0	0	2	missing	1	0	missing	0	5	2	1
184	1	1	2	1	0	0	missing	0	6	3	missing
185	1	1	2	1	missing	missing	missing	0	5	2	missing
186	0	1	2	missing	0	0	0	0	5	2	3
187	1	1	2	1	0	0	0	0	5	2	2
188	0	0	2	missing	1	0	missing	0	5	2	missing
189	0	1	2	1	missing	0	missing	1	5	2	2
190	1	1	2	1	0	missing	0	0	5	2	2
191	0	1	2	1	0	0	missing	0	5	2	1
192	1	1	2	1	missing	0	missing	0	8	3	missing
193	1	1	2	missing	1	0	missing	0	5	2	missing
194	0	1	2	missing	0	0	0	0	5	2	1
195	1	1	2	missing	missing	missing	missing	missing	5	2	missing
196	0	1	2	missing	0	0	0	0	5	2	1

197	0	0	2	missing	missing	missing	missing	missing	3	3	1
198	1	1	2	missing	missing	missing	missing	missing	5	2	missing
199	0	0	2	1	1	0	0	1	5	2	2
200	0	0	2	1	1	0	missing	0	3	3	missing
201	1	1	2	missing	0	0	0	0	5	2	missing
202	1	0	2	missing	missing	missing	missing	missing	5	2	2
203	0	0	2	missing	missing	missing	missing	missing	missing	3	1
204	0	0	2	missing	missing	1	missing	0	5	2	2
205	1	1	2	missing	missing	0	0	0	4	1	2
206	0	0	2	1	missing	0	missing	0	5	2	1
207	0	0	2	missing	missing	0	missing	0	5	2	missing
208	0	0	2	1	1	0	missing	0	5	2	missing
209	1	0	2	missing	1	missing	missing	0	5	2	missing
210	0	1	2	1	1	0	missing	0	5	2	missing
211	1	1	2	missing	0	0	missing	0	5	2	1
212	0	0	2	1	1	missing	missing	0	5	2	missing
213	0	0	2	missing	1	0	missing	0	5	2	1
214	1	1	2	0	0	0	missing	0	5	2	4
215	0	0	2	missing	missing	0	missing	0	3	3	missing
216	1	1	2	missing	missing	missing	missing	missing	missing	3	missing
217	0	0	2	1	1	0	0	0	5	2	3
218	1	0	2	missing	0	0	0	1	4	1	missing
219	0	0	2	0	missing	0	missing	0	5	2	missing
220	1	0	2	1	0	0	missing	0	5	2	missing
221	0	0	2	missing	0	missing	missing	0	5	2	missing
222	0	0	2	missing	missing	missing	0	0	7	3	missing
223	0	1	1	1	0	missing	missing	0	5	2	missing
224	1	1	2	missing	0	0	0	0	5	2	2
225	0	1	1	missing	0	0	missing	1	8	3	missing
226	0	1	2	1	0	0	missing	0	5	2	2
227	0	0	2	missing	1	0	missing	0	5	2	1
228	0	1	2	1	0	0	0	1	5	2	2
229	0	0	2	1	1	0	missing	0	5	2	2

230	0	0	2	missing	missing	0	0	0	5	2	1
231	0	0	2	missing	0	0	0	0	5	2	2
232	1	1	2	missing	0	0	missing	0	5	2	3
233	0	0	2	1	1	0	missing	0	3	3	missing
234	1	1	2	1	0	0	missing	0	5	2	2
235	0	0	2	1	1	0	missing	0	7	3	missing
236	1	1	1	missing	missing	missing	missing	missing	5	2	missing
237	0	0	2	missing	missing	1	missing	1	5	2	missing
238	0	0	2	1	1	1	0	0	6	3	missing
239	0	1	2	1	missing	0	missing	0	5	2	1
240	1	1	2	missing	missing	missing	missing	missing	5	2	2
241	0	0	2	missing	1	0	0	0	5	2	missing
242	1	1	2	1	0	0	missing	0	5	2	1
243	1	0	1	missing	1	missing	missing	0	3	3	missing
244	1	1	2	1	0	0	missing	0	5	2	missing
245	0	0	2	missing	1	0	missing	0	5	2	3
246	0	0	2	missing	1	0	missing	1	5	2	2
247	1	1	2	1	0	0	0	0	5	2	missing
248	1	1	1	missing	0	0	missing	0	5	2	missing
249	0	1	1	missing	missing	0	missing	0	5	2	missing
250	0	0	2	missing	1	0	0	0	5	2	2
251	1	1	1	missing	missing	missing	missing	missing	5	2	missing
252	1	1	2	missing	0	0	missing	0	5	2	1
253	1	0	1	missing	missing	missing	missing	missing	5	2	2
254	1	1	2	1	1	missing	missing	0	5	2	2
255	0	0	1	missing	1	0	missing	0	5	2	missing
256	1	1	1	missing	missing	missing	missing	missing	5	2	missing
257	1	1	2	missing	0	0	missing	0	5	2	1
258	0	0	1	1	missing	0	missing	0	5	2	2
259	0	0	2	1	1	0	missing	1	5	2	2
260	1	1	2	missing	0	0	missing	1	5	2	missing
261	1	0	2	missing	0	0	0	0	3	3	1
262	0	0	2	missing	1	0	0	0	5	2	2

263	1	1	1	1	0	missing	missing	0	4	1	missing
264	0	1	2	0	0	0	missing	0	5	2	missing
265	1	1	2	missing	0	0	1	0	4	1	2
266	0	1	1	missing	0	0	missing	0	2	3	missing
267	1	1	1	missing	missing	missing	missing	missing	missing	3	missing
268	1	1	1	missing	0	missing	missing	0	5	2	1
269	0	1	2	missing	missing	0	missing	0	5	2	1
270	0	0	1	missing	0	0	missing	0	5	2	missing
271	0	0	2	missing	missing	0	missing	0	0	0	3
272	0	0	2	missing	1	0	missing	1	5	2	2
273	0	0	1	missing	missing	0	missing	0	5	2	missing
274	0	0	2	missing	1	0	missing	0	5	2	2
275	1	0	2	missing	1	0	missing	1	8	3	2
276	1	1	1	1	missing	0	missing	0	8	3	missing
277	1	1	1	1	0	0	0	0	5	2	missing
278	0	0	1	missing	1	0	missing	0	6	3	3
279	1	0	1	missing	0	missing	missing	0	8	3	missing
280	0	1	1	1	1	0	missing	0	0	0	3
281	0	0	2	0	1	0	missing	0	5	2	1
282	0	0	1	0	1	0	missing	0	5	2	missing
283	0	1	1	missing	missing	missing	missing	missing	5	2	missing
284	1	1	2	1	0	0	0	1	4	1	missing
285	0	1	1	missing	0	0	0	0	5	2	missing
286	1	1	2	missing	missing	missing	missing	0	1	3	missing
287	0	1	2	1	0	0	0	0	5	2	1
288	0	0	1	1	missing	0	missing	0	4	1	3
289	1	1	1	1	0	0	missing	0	5	2	1
290	1	1	1	missing	0	missing	0	0	5	2	missing
291	1	0	1	1	missing	missing	missing	0	4	1	4
292	0	0	2	missing	missing	0	0	0	5	2	2
293	0	1	2	1	0	0	missing	0	2	3	1
294	0	0	1	missing	1	0	0	0	0	0	missing
295	0	1	1	missing	0	0	missing	0	5	2	1

296	1	1	1	1	0	0	0	0	3	3	missing
297	1	1	1	missing	0	missing	missing	0	5	2	missing
298	1	0	1	missing	1	missing	missing	0	5	2	missing
299	0	0	1	missing	1	0	missing	0	5	2	2
300	0	0	1	missing	1	0	missing	0	5	2	3
301	1	1	1	missing	0	0	missing	0	5	2	1
302	1	1	1	1	missing	missing	missing	0	5	2	missing
303	0	1	1	0	1	missing	missing	1	0	0	missing
304	0	1	2	missing	0	0	missing	0	5	2	2
305	1	0	2	missing	0	missing	missing	0	5	2	2
306	0	0	1	1	0	0	0	0	7	3	3
307	1	1	1	1	0	0	missing	0	5	2	2
308	1	1	1	missing	missing	missing	missing	missing	1	3	missing
309	0	0	1	missing	1	0	missing	1	8	3	2
310	1	1	2	missing	0	0	missing	0	5	2	2
311	1	0	1	1	0	0	missing	0	5	2	2
312	0	1	1	missing	missing	0	missing	0	5	2	2
313	0	0	2	missing	1	0	missing	0	5	2	1
314	1	0	1	missing	1	missing	0	0	5	2	missing
315	0	0	2	missing	1	0	missing	0	2	3	missing
316	0	0	1	missing	missing	missing	missing	missing	5	2	2
317	0	0	1	1	missing	missing	missing	0	5	2	2
318	0	1	1	missing	0	missing	missing	0	5	2	1
319	0	0	1	1	0	0	0	0	5	2	missing
320	0	0	1	missing	1	0	missing	0	5	2	3
321	0	1	1	1	0	0	missing	0	5	2	1
322	1	1	1	missing	missing	0	missing	0	4	1	missing
323	0	1	2	1	0	0	missing	0	5	2	1
324	0	0	1	1	1	0	missing	0	5	2	2
325	0	1	1	1	0	0	0	0	5	2	1
326	0	0	2	missing	1	0	missing	0	5	2	2
327	1	1	1	missing	missing	0	missing	0	3	3	missing
328	1	0	2	missing	1	0	missing	0	5	2	1

329	0	0	1	0	1	missing	missing	0	5	2	missing
330	1	1	1	1	0	0	0	0	5	2	1
331	0	0	1	1	1	missing	missing	0	5	2	missing
332	1	1	1	1	0	missing	missing	0	4	1	4
333	0	0	1	1	1	0	missing	0	5	2	missing
334	1	1	1	1	0	0	0	0	1	3	missing
335	0	0	1	missing	1	0	0	0	8	3	2
336	0	1	2	missing	0	0	missing	0	5	2	1
337	0	1	1	1	missing	missing	missing	0	1	3	missing
338	1	1	1	1	0	missing	missing	0	4	1	4
339	0	0	1	1	1	0	1	0	7	3	2
340	1	1	1	missing	missing	missing	missing	missing	5	2	1
341	1	1	1	1	0	0	missing	0	5	2	missing
342	1	1	1	1	0	0	0	0	5	2	1
343	1	1	1	missing	0	0	missing	0	5	2	1
344	1	1	1	1	0	0	missing	0	5	2	2
345	0	1	1	1	1	0	0	1	7	3	missing
346	0	1	1	1	missing	0	0	0	4	1	2
347	0	0	1	1	missing	0	0	0	5	2	missing
348	0	0	1	1	missing	missing	missing	0	5	2	1
349	1	1	2	missing	1	0	missing	0	4	1	2
350	0	1	1	missing	0	0	missing	0	2	3	missing
351	1	1	1	missing	missing	missing	missing	missing	5	2	2
352	0	0	1	missing	missing	0	missing	0	5	2	missing
353	1	1	1	missing	missing	missing	missing	missing	5	2	missing
354	0	0	1	1	0	0	0	0	5	2	missing
355	0	0	1	1	1	1	missing	1	8	3	3
356	1	1	1	1	missing	missing	missing	0	5	2	missing
357	0	0	1	missing	1	0	0	0	5	2	1
358	1	1	1	missing	missing	missing	missing	missing	5	2	missing
359	1	1	1	1	0	0	missing	0	5	2	missing
360	0	0	1	1	0	0	0	0	5	2	2
361	0	1	1	missing	0	0	missing	0	3	3	2

362	0	1	1	missing	missing	0	missing	0	0	0	2
363	0	0	1	1	0	0	0	0	1	3	missing
364	1	0	1	1	missing	0	missing	0	4	1	missing
365	0	0	1	missing	1	missing	missing	0	5	2	missing
366	0	0	1	missing	1	0	0	0	5	2	3
367	0	1	1	missing	missing	0	missing	0	3	3	1
368	0	0	1	1	1	missing	missing	0	5	2	2
369	1	1	1	missing	missing	missing	missing	missing	5	2	missing
370	0	0	1	missing	missing	missing	missing	missing	5	2	missing
371	1	0	1	missing	missing	missing	missing	missing	5	2	2
372	0	1	1	missing	0	missing	missing	0	5	2	2
373	1	1	1	1	missing	missing	missing	0	4	1	4
374	1	1	1	missing	missing	0	missing	0	8	3	3
375	1	0	1	1	0	0	missing	0	4	1	missing
376	1	0	1	missing	0	missing	missing	0	5	2	1
377	0	1	1	missing	missing	0	missing	0	8	3	missing
378	1	0	1	1	0	missing	missing	0	5	2	2
379	1	0	1	missing	1	missing	missing	missing	missing	3	missing
380	0	0	1	1	1	0	0	0	5	2	missing
381	0	1	1	1	missing	0	missing	0	5	2	missing
382	1	1	1	missing	missing	missing	missing	missing	5	2	missing
383	0	1	1	1	missing	0	missing	0	8	3	missing
384	0	1	1	missing	missing	0	missing	0	5	2	missing
385	0	0	1	1	1	0	missing	0	5	2	2
386	0	0	1	1	0	0	0	0	8	3	missing
387	0	1	1	1	0	0	0	0	5	2	1
388	0	0	1	1	missing	missing	missing	0	5	2	missing
389	0	1	1	1	0	0	0	0	5	2	2
390	1	1	1	missing	missing	missing	missing	missing	5	2	missing
391	1	0	1	missing	missing	0	missing	0	4	1	1
392	0	1	1	missing	0	0	0	0	1	3	missing
393	1	1	1	missing	missing	0	missing	0	5	2	missing
394	1	1	1	missing	missing	missing	missing	missing	5	2	missing

395	1	0	1	missing	0	0	missing	0	5	2	2
396	0	0	1	missing	1	0	missing	0	5	2	1
397	1	0	1	missing	1	0	missing	0	8	3	3
398	0	1	1	missing	missing	0	missing	0	5	2	missing
399	0	1	1	1	0	0	0	0	5	2	2
400	0	0	1	1	missing	0	missing	0	5	2	2
401	0	1	1	1	0	missing	0	0	5	2	2
402	1	1	1	missing	missing	0	1	0	5	2	3
403	1	1	1	1	missing	0	missing	0	4	1	missing
404	0	1	1	1	missing	0	missing	0	5	2	missing
405	1	1	1	1	missing	missing	0	0	5	2	2
406	0	0	1	missing	missing	0	missing	0	5	2	2
407	0	0	1	missing	1	0	0	0	5	2	3
408	0	0	1	1	missing	missing	missing	0	5	2	missing
409	1	1	1	missing	missing	0	missing	0	4	1	2
410	0	1	1	1	missing	0	0	0	5	2	1
411	0	0	1	1	missing	missing	missing	0	5	2	missing
412	0	0	1	missing	1	0	missing	0	5	2	missing
413	1	0	1	missing	missing	missing	missing	missing	5	2	2
414	1	0	1	1	0	0	0	0	4	1	2
415	1	0	1	missing	missing	0	0	0	6	3	4
416	0	0	1	missing	missing	0	missing	0	4	1	missing
417	0	1	1	missing	missing	0	missing	0	5	2	missing
418	1	1	1	1	0	0	0	0	4	1	2
419	0	0	1	0	1	0	0	0	5	2	2
420	1	1	1	missing	1	missing	missing	0	8	3	missing
421	0	0	1	1	1	missing	missing	0	3	3	missing
422	0	1	1	1	missing	0	0	0	1	3	3
423	1	1	1	1	0	missing	0	0	1	3	missing
424	1	0	1	1	1	0	missing	0	5	2	missing
425	1	1	1	1	missing	0	missing	0	5	2	missing
426	0	1	1	missing	0	0	0	0	5	2	1
427	1	1	1	missing	0	0	missing	0	4	1	4

428	0	0	1	1	1	0	missing	0	0	0	3
429	0	0	1	1	0	0	0	0	4	1	missing
430	1	0	1	missing	missing	0	0	0	8	3	missing
431	1	1	1	missing	missing	missing	missing	missing	5	2	missing
432	0	0	1	missing	0	0	missing	1	5	2	2
433	0	0	1	missing	missing	missing	missing	missing	5	2	missing
434	1	1	1	1	missing	0	0	0	1	3	missing
435	1	1	1	0	missing	missing	missing	0	5	2	1
436	0	0	1	1	missing	0	missing	0	5	2	2
437	0	1	1	missing	0	0	missing	0	5	2	2
438	0	0	1	1	missing	0	missing	0	3	3	1
439	1	1	1	missing	1	0	missing	0	4	1	4
440	0	1	1	1	0	0	missing	0	5	2	missing
441	0	1	1	1	0	0	0	0	5	2	missing
442	1	1	1	0	missing	missing	missing	0	5	2	missing
443	0	0	1	missing	1	0	0	0	5	2	missing
444	1	1	1	missing	missing	missing	missing	missing	5	2	missing
445	1	1	1	1	missing	missing	0	0	5	2	missing
446	0	1	1	missing	missing	0	missing	0	4	1	3
447	0	0	1	1	1	0	missing	0	8	3	missing
448	1	1	1	0	1	0	0	0	0	0	1
449	1	1	1	0	0	0	0	0	5	2	missing
450	0	1	1	missing	missing	missing	missing	missing	5	2	3
451	0	1	1	missing	0	missing	missing	0	5	2	missing
452	0	1	1	1	0	missing	missing	1	5	2	missing
453	0	0	1	missing	0	0	missing	0	6	3	missing
454	0	1	1	1	0	0	0	0	5	2	3
455	0	1	1	1	1	0	0	0	5	2	3
456	0	1	1	0	1	0	missing	0	0	0	3
457	0	0	1	missing	1	0	0	0	5	2	missing
458	0	1	0	missing	missing	0	missing	0	1	3	missing
459	0	1	1	1	missing	0	missing	0	5	2	missing
460	0	1	1	missing	missing	0	missing	0	5	2	missing

461	1	1	1	1	0	0	missing	0	4	1	missing
462	0	0	1	0	0	0	0	0	5	2	1
463	1	0	1	missing	0	0	missing	1	2	3	3
464	0	0	1	0	missing	missing	missing	0	0	0	missing
465	0	1	1	0	missing	0	missing	0	5	2	2
466	1	0	1	missing	0	missing	missing	0	4	1	missing
467	0	1	0	1	1	0	missing	0	5	2	missing
468	0	1	0	missing	missing	0	missing	0	5	2	missing
469	0	0	1	missing	0	0	missing	0	0	0	missing
470	1	1	1	missing	missing	missing	missing	missing	5	2	missing
471	0	1	1	missing	missing	missing	missing	missing	5	2	missing
472	0	0	1	0	1	0	0	0	5	2	3
473	0	0	1	missing	missing	0	missing	0	5	2	missing
474	0	1	1	missing	1	0	missing	0	5	2	3
475	0	0	1	0	1	0	0	0	1	3	3
476	0	0	1	missing	0	0	0	0	5	2	2
477	0	1	1	0	0	0	0	0	5	2	2
478	1	1	0	missing	missing	missing	missing	missing	5	2	missing
479	0	0	1	missing	missing	0	0	0	5	2	1
480	0	0	0	missing	1	missing	missing	0	5	2	missing
481	1	1	0	missing	0	missing	missing	0	4	1	missing
482	1	1	1	missing	missing	0	missing	0	4	1	1
483	1	1	1	missing	0	0	missing	0	2	3	3
484	0	0	0	missing	missing	0	0	0	5	2	missing
485	1	0	1	0	1	0	0	0	5	2	missing
486	0	0	1	0	1	0	missing	0	5	2	1
487	0	0	0	1	missing	0	0	0	2	3	missing
488	0	0	0	missing	1	missing	missing	0	5	2	missing
489	1	1	1	missing	0	0	0	0	5	2	missing
490	1	1	1	1	0	missing	0	0	5	2	missing
491	0	0	1	missing	0	missing	missing	0	5	2	missing
492	1	0	1	0	missing	0	missing	0	5	2	2
493	0	1	0	missing	missing	0	missing	0	5	2	missing

494	1	1	1	missing	0	0	missing	0	4	1	missing
495	0	1	0	1	0	0	0	0	3	3	1
496	0	0	0	0	1	0	0	0	0	0	missing
497	1	1	1	missing	missing	0	0	0	4	1	missing
498	0	1	1	1	0	0	missing	0	5	2	2
499	1	1	1	missing	1	0	0	0	5	2	1
500	0	1	1	1	missing	0	missing	0	5	2	2
501	0	1	0	missing	0	missing	missing	0	4	1	2
502	1	1	0	1	0	0	0	0	5	2	missing
503	0	1	0	missing	missing	missing	1	0	5	2	2
504	0	0	1	0	missing	0	missing	0	5	2	1
505	0	1	1	1	missing	0	missing	0	5	2	1
506	0	1	1	missing	missing	0	missing	0	5	2	3
507	0	1	0	missing	missing	missing	missing	missing	5	2	missing
508	1	0	0	missing	missing	missing	0	0	5	2	missing
509	0	1	1	missing	0	0	0	0	5	2	missing
510	1	1	1	missing	0	0	missing	0	5	2	1
511	1	1	1	1	missing	0	missing	0	5	2	missing
512	0	1	1	missing	0	0	missing	0	5	2	1
513	1	1	1	1	missing	0	0	0	5	2	2
514	0	0	1	missing	0	0	missing	0	5	2	1
515	1	1	1	1	0	0	missing	0	5	2	3
516	0	1	1	missing	0	0	missing	0	4	1	missing
517	1	1	0	missing	missing	missing	missing	missing	5	2	missing
518	0	1	0	1	0	0	missing	0	5	2	2
519	1	1	0	0	missing	0	0	0	5	2	missing
520	0	1	1	1	missing	0	missing	0	4	1	missing
521	1	1	0	1	missing	0	missing	0	5	2	missing
522	1	1	1	missing	missing	missing	0	0	5	2	1
523	0	1	0	missing	missing	missing	missing	missing	5	2	missing
524	0	1	1	0	0	0	0	0	0	0	3
525	0	0	0	missing	0	0	0	0	1	3	2
526	0	1	1	1	0	0	missing	0	2	3	1

527	0	1	1	1	missing	missing	missing	0	3	3	1
528	1	0	1	missing	missing	0	missing	0	5	2	2
529	1	1	0	1	0	0	missing	0	5	2	2
530	1	0	1	missing	missing	0	1	0	5	2	2
531	0	1	0	1	missing	missing	missing	0	5	2	1
532	0	1	0	1	0	0	0	0	5	2	2
533	0	0	1	1	missing	0	0	0	4	1	missing
534	0	0	0	1	0	0	missing	0	5	2	1
535	0	1	1	missing	0	0	0	0	5	2	2
536	0	1	1	1	missing	0	0	0	4	1	missing
537	0	0	0	missing	1	0	0	0	5	2	missing
538	1	1	1	missing	0	missing	missing	0	5	2	1
539	0	1	1	missing	missing	0	missing	0	5	2	missing
540	1	1	0	0	missing	missing	missing	0	5	2	missing
541	1	1	0	1	missing	0	missing	0	5	2	missing
542	0	1	1	1	0	0	0	0	8	3	missing
543	1	1	1	missing	0	0	missing	0	5	2	1
544	0	0	1	missing	1	0	missing	0	5	2	2
545	0	1	1	missing	missing	0	missing	0	4	1	3
546	1	1	1	1	0	0	0	0	5	2	1
547	0	1	1	missing	0	0	missing	0	5	2	1
548	0	1	1	missing	0	0	0	0	5	2	4
549	0	0	1	1	0	0	missing	0	8	3	3
550	1	1	0	missing	0	missing	0	0	5	2	2
551	0	1	1	missing	0	0	0	0	4	1	4
552	0	1	0	missing	missing	0	missing	0	5	2	1
553	0	1	0	0	missing	missing	0	0	5	2	missing
554	0	0	1	missing	0	0	missing	0	4	1	3
555	0	1	0	missing	0	0	0	1	5	2	3
556	0	1	0	missing	missing	0	missing	0	5	2	1
557	0	0	0	missing	missing	missing	missing	missing	5	2	2
558	1	0	1	missing	1	0	0	0	5	2	1
559	0	0	0	missing	missing	missing	missing	missing	5	2	missing

560	0	1	0	1	missing	0	missing	0	2	3	3
561	1	0	0	1	missing	missing	missing	0	6	3	missing
562	1	1	1	missing	missing	0	missing	0	5	2	1
563	0	1	0	missing	missing	0	missing	0	4	1	missing
564	0	1	0	1	missing	0	missing	0	5	2	2
565	0	1	1	1	0	0	missing	0	5	2	1
566	0	0	1	missing	0	0	0	0	1	3	1
567	0	0	0	missing	1	0	0	0	7	3	missing
568	0	1	0	0	missing	missing	missing	0	5	2	missing
569	0	1	0	1	0	missing	missing	0	5	2	missing
570	0	1	1	1	missing	0	0	0	5	2	1
571	0	0	1	missing	1	0	missing	0	5	2	1
572	0	1	0	missing	0	0	missing	0	5	2	1
573	0	0	0	1	missing	0	missing	0	6	3	missing
574	0	1	0	1	1	0	0	0	5	2	missing
575	0	0	0	1	0	0	0	0	5	2	2
576	0	1	0	1	0	0	0	0	5	2	missing
577	0	1	0	1	missing	0	missing	0	0	0	missing
578	1	0	0	1	0	0	0	0	5	2	missing
579	1	0	0	missing	1	0	missing	0	0	0	missing
580	0	1	1	1	0	0	missing	0	4	1	1
581	0	1	0	1	0	0	missing	0	8	3	missing
582	1	1	0	missing	missing	missing	missing	missing	5	2	1
583	0	1	0	missing	0	0	0	0	5	2	1
584	0	1	0	missing	0	0	missing	0	0	0	1
585	1	1	0	0	1	0	0	0	0	0	3
586	0	0	0	1	missing	0	0	0	3	3	missing
587	0	1	0	0	0	0	0	0	0	0	1
588	0	1	0	1	0	0	0	0	7	3	1
589	0	0	0	1	0	0	missing	0	6	3	missing
590	1	1	0	missing	0	0	missing	0	5	2	2
591	0	0	0	1	1	0	missing	0	5	2	3
592	0	1	0	missing	0	0	missing	0	0	0	1

593	1	0	0	1	missing	0	missing	0	5	2	missing
594	0	0	0	1	0	0	0	0	5	2	missing
595	0	1	0	missing	missing	0	0	0	5	2	missing
596	0	1	0	missing	1	0	0	0	5	2	missing
597	1	1	0	missing	missing	0	0	0	0	0	1
598	0	1	0	0	missing	0	missing	0	5	2	missing
599	1	0	0	0	missing	0	missing	0	0	0	4
600	0	1	0	missing	missing	0	missing	0	0	0	1
601	0	0	0	1	1	0	missing	0	5	2	1
602	0	0	0	1	missing	0	missing	0	5	2	2
603	1	1	0	missing	missing	0	0	0	1	3	4
604	0	0	0	1	1	0	missing	0	3	3	1
605	0	0	0	0	missing	missing	missing	0	0	0	missing
606	0	0	0	0	0	0	0	0	0	0	missing
607	0	1	0	1	1	0	missing	0	0	0	1
608	0	1	0	1	missing	0	0	0	1	3	missing
609	0	0	0	0	0	0	0	0	5	2	2
610	1	1	0	1	missing	0	missing	0	6	3	missing
611	1	0	0	missing	0	0	missing	0	0	0	3
612	0	1	0	1	0	missing	missing	0	0	0	missing
613	1	1	0	0	0	0	missing	0	0	0	missing
614	0	0	0	0	0	0	missing	0	0	0	1
615	0	1	0	missing	0	0	missing	0	0	0	missing
616	0	0	0	missing	0	0	missing	0	0	0	missing
617	0	1	0	1	0	0	missing	0	4	1	missing
618	0	0	0	missing	0	missing	missing	0	0	0	2
619	0	1	0	0	0	0	missing	0	0	0	missing
620	0	1	0	0	0	0	missing	0	0	0	missing
621	0	0	0	1	missing	missing	missing	0	1	3	missing
622	0	1	0	0	0	0	missing	0	0	0	missing
623	0	0	0	0	0	0	missing	0	0	0	missing
624	0	0	0	0	missing	0	missing	0	0	0	missing
625	0	1	0	missing	0	0	missing	0	0	0	missing

626	0	0	0	0	0	missing	missing	0	0	0	missing
627	0	0	0	0	0	0	missing	0	0	0	missing
628	0	1	0	missing	0	0	missing	0	1	3	2
629	0	1	0	0	0	0	missing	0	0	0	missing
630	0	1	0	0	0	0	missing	0	0	0	missing
631	0	1	0	1	0	missing	missing	0	0	0	missing
632	0	0	0	0	0	0	missing	0	0	0	missing
633	0	1	0	missing	0	0	missing	0	0	0	missing
634	0	0	0	0	missing	0	missing	0	0	0	missing
635	1	1	0	missing	0	0	missing	0	0	0	missing
636	0	1	0	0	0	0	missing	0	0	0	missing

LEGEND

disease site	gender	age, years	BCG	smoking	chronic kidney disease	HIV	diabetes	UN regions	MODIFIED REGION	MTB genotype
0 = PTB	0 = male	0 = 0-24	0 = no	0 = no	0 = no	0 = no	0 = no	1 = Africa	0 = Australia	1 = East Asian
1 = LNTB	1 = female	1 = 25-44	1 = yes	1 = yes	1 = yes	1 = yes	1 = yes	2 = South America	1 = South Asia	2 = Indo Oceanic
		2 = 45-64						3 = Eastern Asia	2 = South East Asia	3 = Euro American
		3 = 65+						4 = Southern Asia	3 = Other	4 = East African Indian
								5 = South East asia		
								6 = Western Asia		
								7 = Europe		
								8 = Pacific islands		
